# Enhancing high-energy powder X-ray diffraction applications using a PILATUS4 CdTe detector

**DOI:** 10.1107/S1600577525000566

**Published:** 2025-02-17

**Authors:** Tilman Donath, Sofia Trampari, Lucas Wagner, Mads R. V. Jørgensen, Frederik H. Gjørup, Stefano Checchia, Marco Di Michiel, Emmanuel Papillon, Gavin Vaughan

**Affiliations:** a DECTRIS Ltd, Täfernweg 1, 5405Baden, Switzerland; b Department of Chemistry and Interdisciplinary Nanoscience Center (iNANO) Aarhus University Langelandsgade 140 8000Aarhus C Denmark; c MAX IV Laboratory Lund University Fotongatan 2 224 84Lund Sweden; d European Synchrotron Radiation Facility 38043Grenoble France; Paul Scherrer Institut, Switzerland

**Keywords:** hybrid photon counting, pixel detectors, cadmium telluride, PILATUS4, time-resolved powder diffraction, *in situ*X-ray powder diffraction

## Abstract

Hybrid photon counting detectors have significantly advanced synchrotron research. In particular, the introduction of large cadmium telluride-based detectors in 2015 enabled a whole new range of high-energy X-ray measurements. This article describes the specifications of the new PILATUS4 cadmium telluride detector and presents results from prototype testing for high-energy powder X-ray diffraction studies conducted at two synchrotrons. The experiments concern time-resolved *in situ* solid-state reactions at MAX IV (Sweden) and fast-scanning X-ray diffraction computed tomography of a battery cell at the ESRF (France). The detector’s high quantum efficiency up to 100 keV, combined with a maximum frame rate of 4000 Hz, enables fast data collection. This study demonstrates how these capabilities contribute to improved time and spatial resolution in high-energy powder X-ray diffraction studies, facilitating advancements in materials, chemical and energy research.

## Abstract 2

Demonstrations are presented of a PILATUS4 CdTe hybrid photon counting detector prototype for high-energy powder X-ray diffraction in materials, chemical and energy research at up to 4 kHz frame rate.

## 1. Introduction

The order-of-magnitude increase in hard X-ray photon flux achieved by fourth-generation synchrotron sources like MAX IV and the ESRF-EBS (Raimondi *et al.*, 2023 ▸; Eriksson *et al.*, 2014 ▸; Tavares *et al.*, 2014 ▸, Tavares *et al.*, 2018 ▸; Liu *et al.*, 2019 ▸) presents an opportunity for even faster high-energy experiments. While hybrid photon counting (HPC) detectors are expected to remain the principal detector technology in the near to mid-term future (Fröjdh *et al.*, 2024 ▸), they are continuously developed further to exploit fully the increased flux of modern synchrotron sources.

HPC detector technology (Brönnimann & Trüb, 2016 ▸) has revolutionized X-ray detection for synchrotron and laboratory sources alike (Förster *et al.*, 2019 ▸). HPC is a direct detection method, achieves high sensitivity and allows the counting of individual photons in each detector pixel, thereby enabling a high dynamic range. HPC detectors have zero readout noise and add no background to the signal, which enables measurements with high signal-to-noise ratio (SNR). Their compatibility with high flux, and the high SNR and dynamic range they provide, present a significant advantage over traditional CCD, CMOS and flat-panel detectors in demanding scientific and industrial applications. Their high frame rate capability makes HPC detectors ideal for applications requiring high temporal resolution. The availability of cadmium telluride (CdTe) as a sensor material (Pennicard *et al.*, 2017 ▸; Šišak Jung *et al.*, 2017 ▸) has made it possible to extend the advantages of HPC technology to conduct research on high-energy X-ray beamlines, overcoming the limitations in speed and dynamic range of the flat-panel and image-plate detectors commonly used previously.

Today, the CdTe detectors of the PILATUS3 and EIGER2 series produced by DECTRIS are successfully applied in a wide variety of high-energy X-ray techniques and research fields, including time-resolved and *in situ* powder X-ray diffraction (PXRD) (Schultheiß *et al.*, 2018 ▸; Lukin *et al.*, 2017 ▸) and diffraction tomography (Vamvakeros *et al.*, 2016 ▸; Finegan *et al.*, 2019 ▸), pair distribution function analysis (Grünewald *et al.*, 2022 ▸; Cerantola *et al.*, 2023 ▸), analyzer-based high-resolution PXRD (Fitch *et al.*, 2023 ▸), various high-pressure experiments (Tschauner *et al.*, 2018 ▸; Prakapenka *et al.*, 2021 ▸; Mezouar & Mathon, 2024 ▸), material studies of texture and microstructure (Yuan *et al.*, 2018 ▸), and three-dimensional X-ray diffraction (3DXRD) (Ball *et al.*, 2022 ▸).

PILATUS4 detectors of different sizes and for low- and high-energy applications, using Si and CdTe sensors, respectively, are under development at DECTRIS Ltd. Here, we describe the PILATUS4 CdTe synchrotron detector and report on two high-energy PXRD experiments conducted at MAX IV and the ESRF-EBS using a two-module detector prototype. These experiments demonstrate its capabilities in time-resolved and scanning-type applications within materials, chemical and energy research.

## 2. PILATUS4 CdTe detector

The PILATUS4 is an HPC detector (Brönnimann & Trüb, 2016 ▸) built on a readout chip (PILATUS4 ASIC) with a pixel size of 150 µm. Individual detector modules of 513 × 255 pixels (77 mm × 38 mm) allow the assembly of multi-module detectors with a large active area. The parameters of the PILATUS4 CdTe are described in Table 1 ▸.

**Figure 1:**
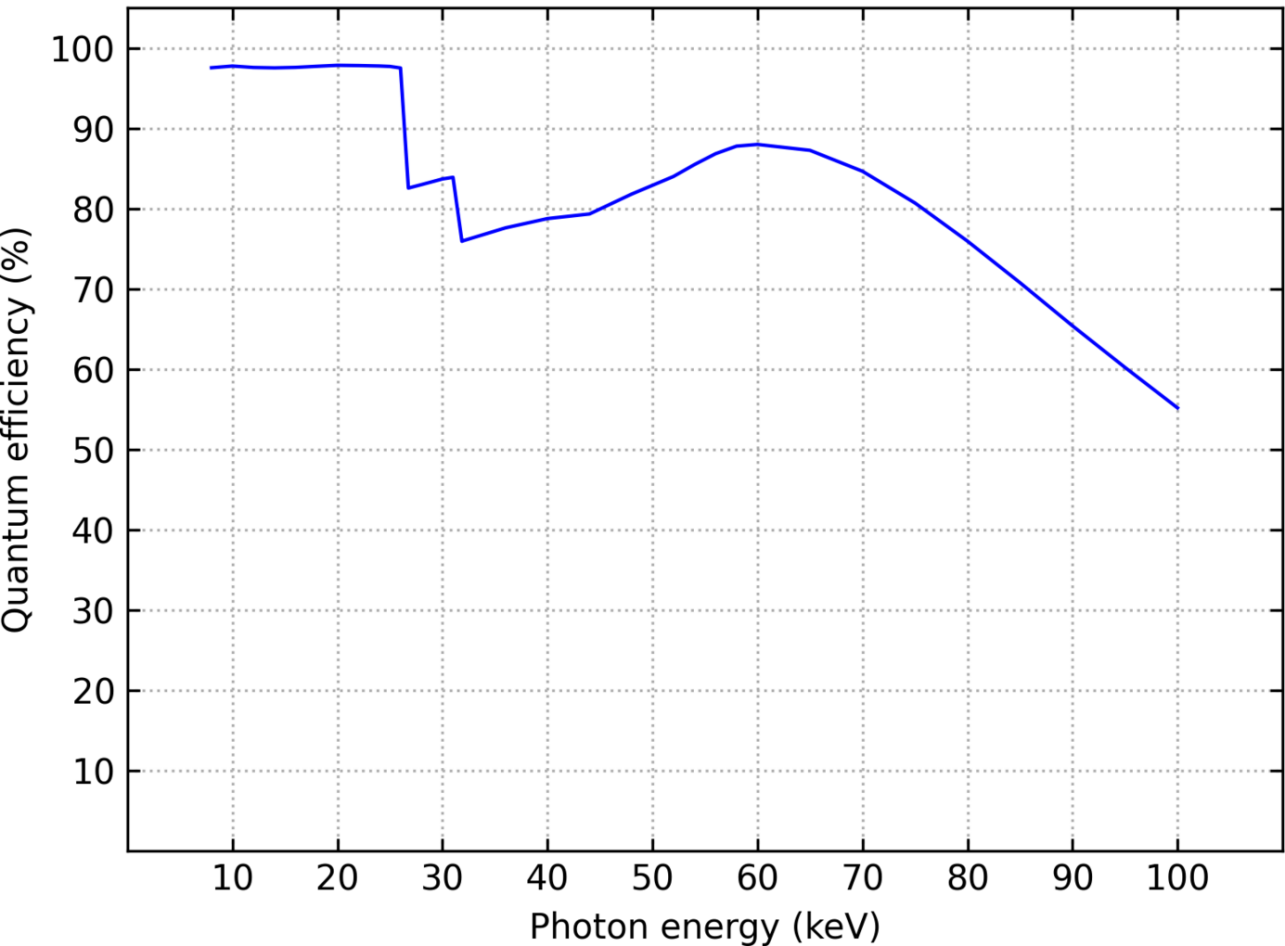
Quantum efficiency of PILATUS4 CdTe for photon energies in the range 8 keV to 100 keV, with threshold set to 50% of the photon energy. The two jumps in the QE data at the *K* absorption edges of Cd (26.7 keV) and Te (31.8 keV) are caused by fluorescence escapes occurring for photon energies above the absorption edges. The QE was determined using a Monte Carlo simulation code (Trueb *et al.*, 2017 ▸).

The detector uses a 1 mm-thick CdTe sensor, resulting in high absorption efficiency over a wide range of photon energy. Fig. 1 ▸ shows the quantum efficiency (QE, the fraction of detected photons relative to the number of incident photons) of the PILATUS4 CdTe over the range 8–100 keV. QE is above 75% up to 80 keV, then decreases at higher energies but stays above 50% at 100 keV.

PILATUS4 can acquire images at a frame rate of up to 2000 Hz, and up to 4000 Hz in an 8-bit readout mode. Depending on the selected frame rate, the detector automatically sets the required image bit depth to 32 bit (using internal auto-summation), 16 bit or 8 bit, which determines the accessible dynamic range; this is described in more detail by Donath *et al.* (2023 ▸).

Another characteristic of the detector is the continuous readout, which is enabled by using two counters per threshold. One counter is read out while the other one is activated for counting. Thereby, the dead time between two successive exposures is only 100 ns, which is the time needed for switching the two counters and is equivalent to a maximum of 0.04% of the exposure time (at the maximum frame rate of 4 kHz). This corresponds to a duty cycle defined as (counting time) / (counting time + switching time) of 99.96% or higher. A high duty cycle and high QE ensure efficient use of the incident radiation, which is essential for kilohertz data collection.

High count rates are enabled by the instant-retrigger unit in the PILATUS4 ASIC, which realizes a form of non-paralyzable counting (Loeliger *et al.*, 2012 ▸; Zambon, 2021 ▸), resulting in count rates above 10^7^ counts s^−1^ pixel^−1^. The shortest possible exposure time of about 100 ns is supported by electronic gating (using internal or external sources), even though it was not employed in the tests presented here.

Four energy thresholds can be independently adjusted to deliver up to four images with different spectral information. A threshold set to 50% of the primary photon energy allows optimal signal detection while avoiding any pixel cross talk. Setting the threshold closer to, but still below, the primary photon energy supresses the fluorescence background from the *K* and *L* edges of most elements, while simultaneously a threshold set above the photon energy can be used to suppress higher-energy signals, such as higher harmonics. The calibrated threshold range goes up to 80 keV, making a wide range of elements accessible for fluorescence suppression.

The control and data readout interfaces of PILATUS4 are adapted from the EIGER2 detectors (Donath *et al.*, 2023 ▸; Burian *et al.*, 2023 ▸) and have been enhanced with functionality to support four energy thresholds.

The system offers two high-performance data readout interfaces accessible through the detector control unit (DCU), which is a dedicated high-performance server. Continuous data acquisition is facilitated via two 100 GbE network connections for high-throughput data transfer, enabling data retrieval as HDF5 files or via ZeroMQ data streams. This architecture allows beamlines to perform long-duration image acquisition, without being restricted to burst-mode operation. This capability is essential for scans extending over minutes to hours, such as XRD tomography or *in situ*/*operando* experiments, and it is indispensable for capturing non-repeatable time-resolved experimental data.

## 3. PXRD demonstration experiments

### 3.1. PILATUS4 detector prototype for testing

**Figure 2:**
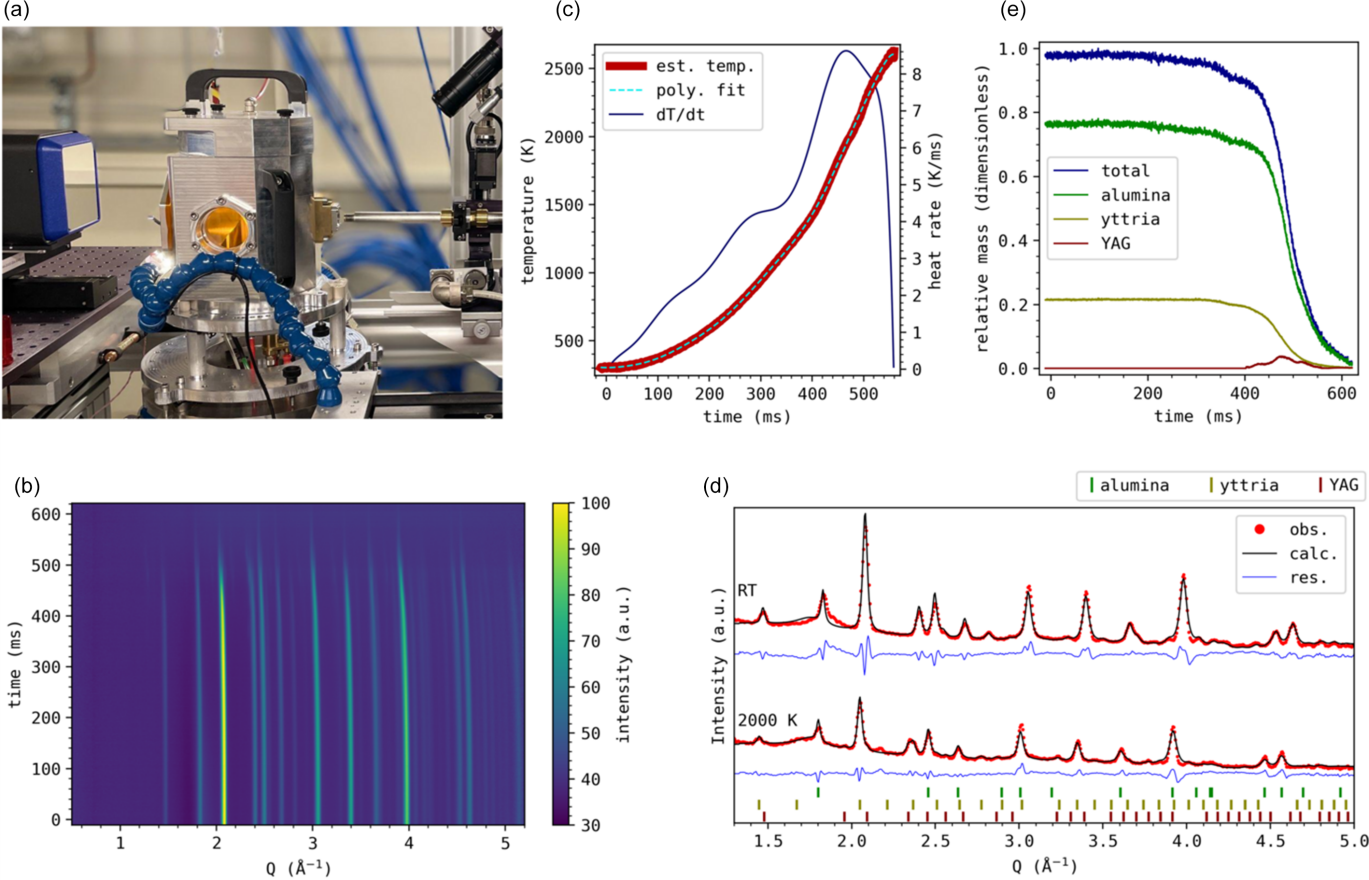
(*a*) Photograph of the setup on the MAX IV beamline DanMAX. The ARΩS furnace (center, with orange polyimide windows) is on a hexapod stage in front of the PILATUS4 CdTe detector prototype (left: black with blue frame and reflective entrance window), with the X-ray beam coming from the right. (*b*) Heat map of the integrated diffraction data as a function of time, background corrected. (*c*) Estimated sample temperature and heat rate determined from refined alumina lattice parameters. (*d*) Selected Rietveld refined patterns at room temperature and 2000 K. (*e*) Evolution of relative masses determined from the refined scale factors.

For the PXRD experiments, a PILATUS4 detector prototype consisting of two detector modules with an approximately square active area of 77.0 mm × 79.5 mm (513 × 530 pixels) was used, as shown in Fig. 2 ▸. This prototype detector was able to operate at up to 4500 Hz frame rate, which corresponds to the technical bandwidth limit for PILATUS4 detectors of all sizes (see footnote to Table 1 ▸).

### 3.2. Enhancing time resolution in PXRD study of solid-state reaction *in situ*

The capability of PILATUS4 for studying a time-resolved reaction *in situ*, near the maximum frame rate of the detector, was demonstrated on the DanMAX beamline at the MAX IV synchrotron (Lund, Sweden). This beamline offers flexible environments for high-energy diffraction experiments under *in situ* and *operando* conditions.

Solid-state reactions, such as sintering and calcination, are perhaps the most fundamental synthesis methods in materials science. Such reactions are typically associated with hour-long high-temperature methods, mainly due to the slow reaction rates and diffusion lengths in the solid state, but also to a great extent due to large furnaces with slow heating rates. Recent *in situ* ultrafast high-temperature sintering (UHS; Wang *et al.*, 2020 ▸) experiments performed on DanMAX have shown reaction times of the order of seconds for both sintering and calcination of nano-sized iron oxides. This is in stark contrast to otherwise long reaction times normally associated with such solid-state reactions (Shyam *et al.*, 2023 ▸; Laursen *et al.*, 2024 ▸).

In this experiment, we are interested in studying the archetypical solid-state reaction between yttria, Y_2_O_3_, and alumina, Al_2_O_3_, forming yttria aluminium garnet (YAG), Y_3_Al_5_O_12_, but under UHS conditions. A mixture of Al_2_O_3_ and Y_2_O_3_ was pressed into a pellet and placed in the ARΩS furnace (Shyam *et al.*, 2023 ▸) shown in Fig. 2 ▸(*a*). The furnace uses resistive heating of a carbon felt filament surrounding the samples and is designed for very fast heating rates. The furnace was run at maximum power to make full use of the high acquisition rate of the PILATUS4 detector.

The beam was monochromated using a horizontally deflecting double multilayer monochromator to an energy of 25.09 keV with a Δ*E*/*E* of approximately 1%. The geometry of the setup was refined using an LaB_6_ standard (SRM 660c, NIST). The beam was attenuated by approximately 30% to a flux of approximately 2 × 10^14^ photons s^−1^ to keep the number of counts per frame (of 220 µs exposure time) within the 8-bit range available at the detector’s highest frame rate. This corresponds to a bit-depth limited maximum count rate of 255 counts / 220 µs = 1.16 × 10^6^ counts s^−1^ per pixel in the 8-bit mode. The diffraction images were integrated directly using the ZeroMQ stream using the *MATFRAIA* algorithm (Jensen *et al.*, 2022 ▸).

A heat map of the azimuthally integrated diffraction data is shown in Fig. 2 ▸(*b*) for a selected time interval (2750 frames, 632 ms), starting just before the heating was initiated at *t* = 0. As the sample heats, the peaks shift to lower *Q* values due to thermal expansion. Between 400 ms and 500 ms faint peaks of the YAG phase emerge, just before the sample melts at ∼550 ms. The diffraction patterns are of sufficient quality to perform sequential Rietveld refinement, mainly limited by parasitic peaks from minor impurities and from the sample environment (graphite filament and polyimide windows). The actual sample temperature can be estimated by comparing the refined lattice parameters of alumina with tabulated thermal expansion coefficients (Munro, 1997 ▸), as shown in Fig. 2 ▸(*c*), which also shows the estimated heating rate based on the derivative of an arbitrary 12-degree polynomial fit of the temperature. While not exact, it provides some insight into the sample temperature, which would be difficult to determine with conventional thermocouples or pyrometric cameras due to the high temperatures (>2500 K), the enclosed nature of the sample environment and the high heating rates (almost 10 000 K s^−1^). Fig. 2 ▸(*d*) shows two selected Rietveld refined diffraction patterns, one at room temperature and one at ∼2000 K. The figure shows decent Rietveld fits of the three main phases: alumina (α-Al_2_O_3_), yttria (Y_2_O_3_) and YAG (Y_3_Al_5_O_12_), sufficient to determine the alumina lattice parameters and perform an overall quantitative analysis. Fig. 2 ▸(*e*) shows the evolution of the relative masses of the three primary phases, calculated from the refined scale factors weighted by the mass and unit-cell volume of each phase. The quantitative analysis corroborates the brief emergence of the YAG phase observed in Fig. 2 ▸(*d*), correlated with a decrease in the alumina and yttria content. The appearance of the YAG phase is followed shortly afterwards by the melting of all three phases, with alumina the last phase to melt in accordance with the alumina–yttria phase diagram at the given composition. The relative mass of YAG starts decreasing at ∼470 ns, followed by an increase shortly before melting. We attribute this to the sample moving as it starts melting, leading to different parts of the sample being probed by the X-ray beam.

The UHS conditions are highly interesting for the synthesis of products with volatile elements that evaporate during conventional high-temperature synthesis. The fast processing will, due to less evaporation, allow better control of the stoichiometry. The DanMAX beamline currently uses a PILATUS3 X CdTe 2M for all diffraction experiments, and a significant fraction are conducted at 250 Hz. The dead time using the PILATUS3 at this speed is 25%, which is nearly fully eliminated using a PILATUS4. The 18-fold higher acquisition rate achieved with the PILATUS4 detector compared with the current detector (4.5 kHz versus 250 Hz) increases the temperature resolution accordingly. For an average heating rate of ∼7300 K s^−1^ when the chemical reaction occurs (time range 400–550 ms), the temperature variation during the capture of each diffraction image is reduced from ∼30 K, possible with the current detector, to ∼2 K. This facilitates *in situ* studies of chemical reactions at high heating rates. As demonstrated above, the intensity available on modern beamlines enables *in situ* experiments in the kilohertz range; this is not possible using the old detector but is readily available with the new PILATUS4 detector, which will help to obtain structural data on relevant time scales.

### 3.3. Increasing resolution in *operando* XRD-CT on batteries

The ultra-fast collection of X-ray diffraction computed tomography (XRD-CT) of a large-format Li-ion battery was performed on the ID15A beamline (Vaughan *et al.*, 2020 ▸) of the ESRF (Grenoble, France). The detector was operated at a 4 kHz frame rate here, showcasing its use for such scanning-based applications, and compared against acquisition at 200 Hz as previously performed with the PILATUS3 X CdTe 2M detector currently installed on the beamline.

The battery was a discharged LG Chem INR-18650-MJ1 commercial cell (Nkon, Netherlands) containing an NMC811 (LiNi_0.8_Mn_0.1_Co_0.1_O_2_) cathode and a graphite–silicon anode, and mounted with its long axis in the center of rotation of the ID15A diffractometer. Inside the battery there is a cylindrical ‘jellyroll’ structure, consisting of alternating layers of cathode (typically 100 µm thick), separator (15 µm), anode (80–100 µm) and current collectors (10 µm) (Heenan *et al.*, 2020 ▸). XRD-CT measurements took place on the ID15A beamline at the ESRF synchrotron (Grenoble, France), taking advantage of the 30-fold increase in high-energy photon flux density after the EBS upgrade (Raimondi *et al.*, 2023 ▸). The X-ray wavelength was λ = 0.1240 Å (100 keV), the sample-to-detector distance was 684 mm, and the X-ray beam was focused to either a 200 µm or to a 32 µm horizontal size depending on the target spatial resolution. Collecting data from a 2D section of the 18650-MJ1 battery took about 200 s (including 10 s for starting/stopping motors), either at a 200 Hz frame rate and 200 µm resolution or at a 4 kHz frame rate and 32 µm resolution. During XRD-CT measurements the battery was continuously rotated while being scanned horizontally across the X-ray beam in steps defined by the horizontal beam size. The step size in turn determines the spatial resolution of the reconstructed tomogram. To obtain each tomogram, the raw diffraction images collected at all rotation and translation positions were integrated using the *pyFAI* software (Ashiotis *et al.*, 2015 ▸). Integrated data were corrected for incident flux and sample attenuation, then reconstructed using a filtered back projection (FBP) algorithm.

Previous work (Heenan *et al.*, 2023 ▸) on the same 18650-MJ1 battery, using a PILATUS3 detector, showed XRD-CT data collected during electrochemical operation. In order to acquire multiple tomograms during high-rate discharges (3C, or 20 minutes for a full discharge), the authors selected a spatial resolution of 200 µm, which enabled them to resolve the internal jellyroll structure of the battery while affording a time resolution of 200 s at a 200 Hz frame rate. A maximum available 4 kHz frame rate offers two options: one is to keep the same 200 µm spatial resolution and scan the 18650 battery in about 30 s; the other is to keep the scan time constant (200 s) and scan across the battery with a smaller beam, thereby increasing the spatial resolution. The first case can be best applied to *operando* experiments studying high-rate battery operation, in which sub-minute scans improve the sensitivity to transient structural effects such as non-equilibrium lithiation stages in both electrodes. In the second case, the increased resolution of the spatial maps enables accurate localization of structural/chemical inhomogeneities within individual electrode layers while affording adequate temporal sampling of the charge/discharge curve.

**Figure 3:**
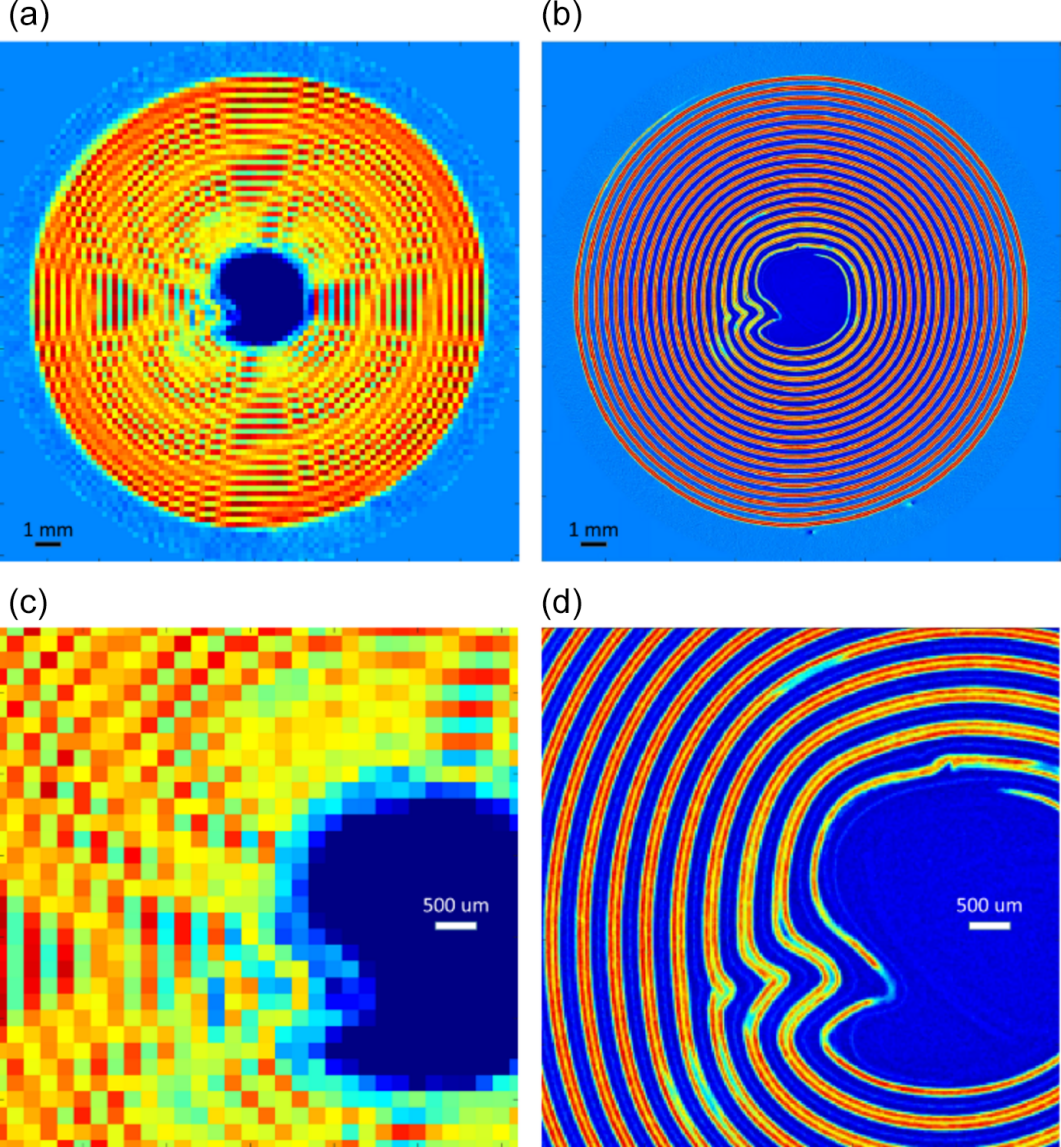
XRD-CT reconstructions showing intensity distributions of the (003) reflection of the main phase related to the NMC cathode. Panels (*a*) and (*c*) show the full map and a detail, respectively, obtained at 200 µm resolution and 200 Hz frame rate. Panels (*b*) and (*d*) show the full map and a detail, respectively, obtained at 32 µm resolution and 4000 Hz frame rate.

Fig. 3 ▸ compares heat maps of the NMC (003) peak intensity measured at a 200 Hz frame rate and 200 µm resolution [panels (*a*) and (*c*)] against 4 kHz and 32 µm resolution [panels (*b*) and (*d*)]. In the lower-resolution data, each period of the jellyroll is described on average by 2 pixels. Therefore, the reconstructed diffraction patterns associated with each pixel contain intensity from two electrode layers, the adjacent current collectors and the separator. In the higher-resolution data, each electrode layer is described by 3 or 4 pixels. Hence, the reconstructed patterns not only avoid overlap with other phases (*e.g.* from the counter-electrode or current collectors), but their further analysis can be used to describe structural gradients within the electrode layer. While large pixels necessarily capture varying amounts of cathode phase together with other parts of the layer, the high-resolution data set permits isolation of the NMC phase, even along the jagged edges of the jellyroll.

**Figure 4:**
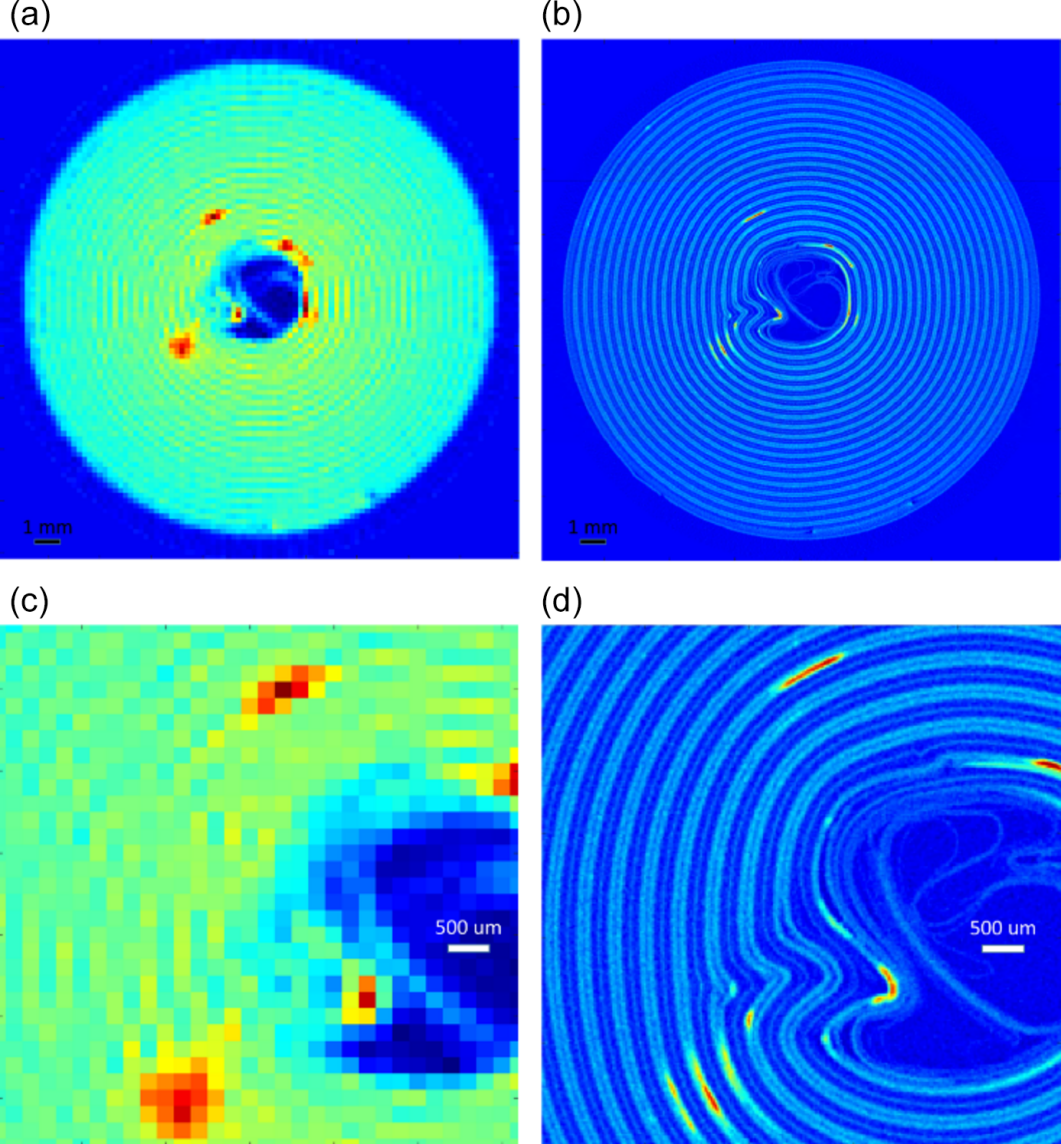
XRD-CT reconstructions showing intensity distributions of the main Bragg peak of a degraded NMC phase formed due to long-term cycling of the battery. Panels (*a*) and (*c*) show the full map and a detail, respectively, obtained at 200 µm resolution at 200 Hz frame rate. Panels (*b*) and (*d*) show the full map and a detail, respectively, obtained at 32 µm resolution at 4000 Hz frame rate.

Internal deformation of this battery was caused by prolonged cycling, especially at high rates of discharge. As reported by Heenan *et al.* (2023 ▸), the battery had previously undergone over 1200 cycles of charge–discharge, and so one of the goals of the XRD-CT characterization was to identify byproduct phases originating from various degradation mechanisms. As a qualitative example, Fig. 4 ▸ shows heat maps of the main Bragg peak intensity of a secondary phase originating from the degradation of the NMC811 cathode. Both low- and high-resolution data show that the secondary phase is localized in certain layers and at definite azimuthal positions, but only the high-resolution data [panels (*b*) and (*d*)] permit intra-layer localization and accurate determination of phase fractions and lattice parameters. A detailed analysis of these structures will be presented in a later publication.

## 4. Discussion and conclusions

The above experiments demonstrate the benefits PILATUS4 can bring in high-energy synchrotron PXRD for research in chemistry, materials and engineering science. PILATUS4 detectors offer the key advantages of very high frame rates while offering high overall detection efficiency. Compared with the PILATUS3 CdTe 2M detector currently installed on both test beamlines, it increases the maximum frame rate from 250 Hz to 4 kHz and eliminates the readout time of 0.95 ms between frames.

A significant benefit of the PILATUS4 detector series is that the high frame rates are available on large-area detectors. While the presented measurements were conducted with a relatively small prototype detector consisting of two modules, the larger PILATUS4 detectors up to 32 modules (4 Mpixels) size achieve the same high frame rate of 4 kHz. With larger detectors it is often possible to place the detector at a larger sample-to-detector distance (SDD), resulting in several advantages over smaller detectors: (i) the detector can be further away from *in situ* setups, (ii) the number of photons is distributed over more pixels, thereby alleviating the need for beam attenuation, and (iii) a better signal-to-background ratio is often achieved, *e.g.* as fluorescence and air scattering are reduced by approximately 1/SDD^2^, while the diffraction signal is only reduced by approximately 1/SDD.

For time-resolved measurements, such as the calcination study above, the high frame rate of up to at least 4 kHz and the suppressed readout time directly determine the accessible time resolution (250 µs or below). The ability to acquire images at this high frame rate *continuously* (in contrast to being limited to acquiring burst sequences) enables processes to be measured and followed over extended periods, *e.g.* to select retrospectively the relevant moment of a reaction from a long measurement.

For scanning-based techniques, as shown in the XRD-CT example above, the increase in the frame rate allows an increased spatial sampling rate, and hence higher spatial resolution without increasing the measurement time. Obviously, the higher speed can alternatively enable a reduction in the measurement time, thereby allowing larger volumes or a larger number of samples to be scanned, or the scan frequency in *operando* measurements to be increased. Hence, scientists can perform more detailed studies across a wider range of experimental conditions.

## Figures and Tables

**Table 1:** PILATUS4 CdTe detector parameters

Pixel size	150 µm × 150 µm
No. of pixels per ASIC	128 × 128
Maximum pixel count rate	>10^7^ photons s^−1^ pixel^−1^
Thresholds, counters per pixel	Four low-energy discriminators, eight 16-bit counters (two per threshold)
Maximum frame rate	2000 Hz in 16 bit-mode, 4000 Hz in 8-bit mode†
Readout time	0.1 µs (dead time of continuous readout, see text)
Image bit depth	32, 16 or 8 bits
Sensor type	CdTe, 1 mm thick
Threshold range	4–80 keV
Control and data readout interface	HTTP REST-like API (‘SIMPLON’) via 2× Duplex fiber optics 100 GbE connection (HDF5 files, ZeroMQ data stream and TIFF monitor)
Data compression	BSLZ4 (default), LZ4

† These maximum frame rates are achieved for all three active areas planned for synchrotron application (1, 2 and 4 Mpixels). This is as a minimum design goal, while technically possible are 2250 Hz in 16-bit and 4500 Hz 8-bit readout modes.

## Data Availability

Data and results can be shared upon request.
